# Nucleolar protein NOP2 could serve as a potential prognostic predictor for clear cell renal cell carcinoma

**DOI:** 10.1080/21655979.2021.1960130

**Published:** 2021-08-01

**Authors:** Gang Wang, Fangfang Qu, Shouyong Liu, Jincai Zhou, Yi Wang

**Affiliations:** a Department of Urology, The Affiliated Jianhu Hospital of Nantong University, Jiangsu Province, China; b Department of Anesthesiology, The Affiliated Jianhu Hospital of Nantong University, Jiangsu Province, China; c Department of Urology, The First Affiliated Hospital of Nanjing Medical University, Nanjing, Jiangsu Province, China; d Department of Urology, Affiliated Hospital of Nantong University, Nantong, Jiangsu Province, China

**Keywords:** NOP2, clear cell renal cell carcinoma, prognosis, TCGA

## Abstract

As an indispensable part for cancer precision medicine, biomarkers and signatures for predicting cancer prognosis and therapeutic benefits were urgently required. The purpose of this study was to investigate the prognostic roles of NOP2 in renal clear cell carcinoma (ccRCC) for overall survival (OS) and its relationships with immunity. NOP2-related gene expression matrix associated with clinical information was obtained from the Cancer Genome Atlas (TCGA) ccRCC dataset and NOP2-related pathways were identified by gene set enrichment analysis (GSEA). Associations among the NOP2 expression and MSI, TMB, TNB, and immunity were also explored. Both the NOP2 mRNA and protein/phosphoprotein had a higher expression in ccRCC tumor tissues than in normal kidney tissues (both *P* < 0.001) and elevated NOP2 expression was associated with poor OS (*P* < 0.001). Logistic regression analysis revealed the NOP2 expression was significantly linked to stage, age, grade, N stage, T stage, and M stage (all *P* < 0.05). Univariate/multivariate Cox hazard regression analysis results indicated that NOP2 was an independent prognostic factor for OS in ccRCC and GSEA revealed five NOP2-related signaling pathways. Nomogram based on NOP2 and eight clinical characteristic parameters (grade, age, stage, gender, T stage, race, M stage, N stage) was constructed and carefully evaluated. Furthermore, NOP2 gene expression was also found to be significantly related to MSI, TMB, and immunity. Our findings revealed that NOP2 might be a potential prognostic factor for OS in ccRCC and it was significantly associated with immunity, MSI, and TMB.

## GRAPHICAL ABSTRACT

**Figure:**
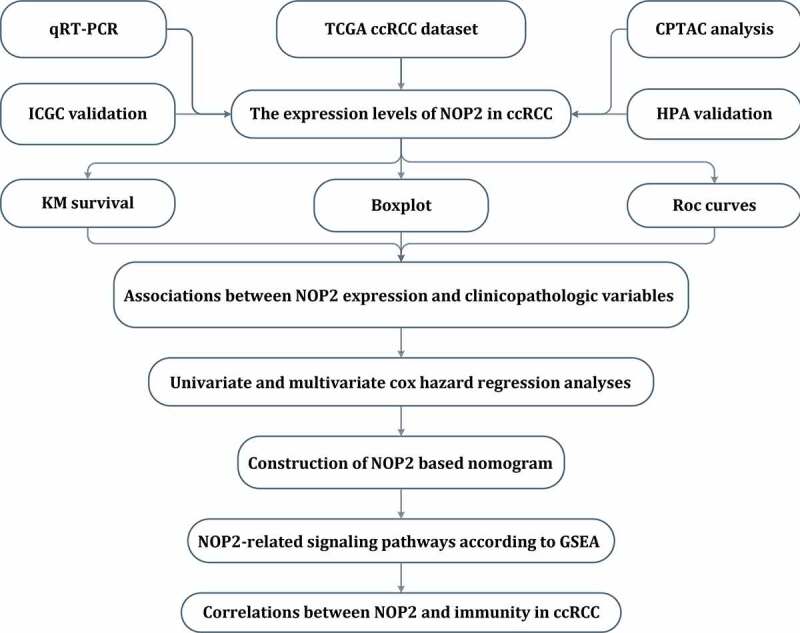

## Introduction

According to the cancer statistics, renal cell carcinoma (RCC) is one of the most common solid malignancy diagnosed in the genitourinary system, and there will be 76,080 newly estimated cases as well as 13,780 newly estimated deaths in the USA, 2021 [1]. Among them, clear cell renal cell carcinoma (ccRCC) accounts for almost 80% of all primary kidney tumors and is the most common form of RCC [2]. Currently, surgical treatment remains the mainstay of therapy for RCC; however, there are still 20%–30% cases developing metastatic disease after surgery and the RCC patients’ 5-year survival rate is about 55% [3,4]. Moreover, biological tumor activity of RCC ranges from indolent to highly lethal and this could also result in widely varied treatment outcomes [5]. Hence, it is urgent to understand RCC tumor biology and explore prognostic biomarkers for these patients.

Human NOP2 nucleolar protein (also known as NOL1, p120, NSUN1, or NOP120) is a member of the NOP2/NSUN RNA-methyltransferase family, also containing six other genes (NSUN2-7) [6]. As reported by Ma et al., NOP2 was highly expressed in various cancer types and it could promote mouse fibroblast growth as well as tumor formation [7]. Sun et al. revealed that LncRNA PVT1 could promote prostate cancer metastasis through targeting tumor suppressor microRNAs and then increase NOP2 expression [8]. Moreover, the LINC00963/miR-542-3p/NOP2 axis could act as an inducer of prostate cancer metastasis and have a diagnostic and therapeutic potential for these patients [9]. Therefore, NOP2 was currently regarded to be a potential biomarker for cancer aggressiveness [10]. However, no researcher had explored the roles of NOP2 in ccRCC. Hence, it was the first time for us to explore its potential roles in ccRCC by means of the Cancer Genome Atlas database (TCGA) data mining.

Thanks to the developments in microarray technology and high-throughput sequencing, we nowadays have the ability to identify the key genes related to tumor prognosis and progression by means of bioinformatics analysis [11]. Based on these, a lot of prognostic markers have been identified and various prognostic models have been established. All of these have been widely utilized to inform disease prognostic stratification and discover novel drug targets [12–14]. By means of TCGA data mining, we obtained a lot of potential biomarkers for ccRCC. We further matched the roles of these biomarkers in other datasets to reduce the scope and to make our results more persuasive. Moreover, after searching in PubMed (https://pubmed.ncbi.nlm.nih.gov/), no researcher had revealed the roles of NOP2 in ccRCC. Hence, this article was intended to investigate the prognostic roles of NOP2 in ccRCC for overall survival (OS) and its relationships with immunity. Our results were anticipated to provide a promising candidate therapeutic method in ccRCC for future targeted treatment.

## Materials and methods

### Acquisition of single gene expression matrix

Gene expression profile matrix and clinical information of ccRCC were downloaded from TCGA (https://tcga-data.nci.nih.gov/tcga/; accessed on March 1, 2021), involving 72 normal and 539 tumor tissues. All gene expression profile matrix was standardized by R software (version 3.5.1; https://www.r-project.org/) [15] and we further did an overlap with NOP2 mRNA to get NOP2-related single-gene expression and clinical information. In addition, OS was selected as the main outcome of this study and ‘limma’ package was utilized to calculate differently expressed genes (DEGs), under the threshold of adjusted P-value (FDR) <0.05 as well as |log2 fold change|≥1.

### Immunohistochemical staining in HPA database, total protein, or phosphoprotein expression by CPTAC analysis

The Human Protein Atlas (HPA, http://www.proteinatlas.org/) online database was explored to validate the NOP2 protein expression in ccRCC by immunohistochemical staining by HPA040119 antibody [16]. We also utilized the UALCAN database (http://ualcan.path.uab.edu/analysis-prot.html) to validate the NOP2 total protein expression between the primary ccRCC tumor and normal tissues by CPTAC analysis [17]. Moreover, NOP2 with phosphorylation sites at the S58, S177T181, T191 and S728 were also explored the differences between the primary tumor and normal tissues [18].

### Quantitative real-time PCR (qRT-PCR)

Ten pairs of clinical ccRCC samples and adjacent kidney tissues were obtained from primary ccRCC patients undergoing radical nephrectomy at the Department of Urology, Affiliated Jianhu Hospital of Nantong University. According to the manufacturer’s instructions, total RNA was extracted using TRIzol reagent, cDNA was synthesized and the qRT-PCR was performed and calculated by means of 2^−ΔΔCt^ methods. Related primers were displayed as following: NOP2, F: 5ʹ- AAGGGTGCCGAGACAGAACT-3ʹ, R: 5ʹ-GAGCACGACTAGACAGCCTC-3ʹ; β-actin, F: 5ʹ-CTCGCCTTTGCCGATCC-3ʹ, R: 5ʹ-TTCTCCATGTCGTCCCAGTT-3ʹ. This study was approved by the Institutional Research Ethics Committees of The Affiliated Jianhu Hospital of Nantong University.

### Genetic alteration analysis by cBioPortal for cancer genomics

We searched the cBioPortal for Cancer Genomics (http://cbioportal.org) database to explore the NOP2 alteration frequency, CNA (copy number alteration) and mutation type in all TCGA tumors via TCGA pancancer atlas studies by querying NOP2 gene. Moreover, the disease-specific, overall, progression-free and disease-free survival differences for ccRCC with or without NOP2 genetic alteration were also presented by K-M plots with log-rank p-values via KIRC (TCGA, Pancancer Atlas) by querying NOP2 gene [19,20].

### Univariate/multivariate Cox hazard regression analyses and nomogram construction

To obtain independent prognostic factors for ccRCC, we utilized univariate/multivariate Cox hazard regression analyses to exclude NOP2 and eight clinical characteristic factors (age, grade, stage, gender, race, T stage, M stage, N stage) with little OS values by R package (version 3.5.1; https://www.r-project.org/). To predict OS probabilities, the R ‘rms’ and ‘survivalROC’ package was performed to create a nomogram and calculate the AUC values between individual predictors and survival rate. Moreover, the calibration curves and C-index were also utilized to assess the performance of the constructed nomogram.

### Gene set enrichment analysis (GSEA) and protein–protein interaction (PPI) network analysis

In order to identify NOP2-related signaling pathways, GSEA was performed to figure out the gene sets displayed statistically significant differences among high-NOP2 groups and low-NOP2 groups, with the consideration of the normalized enrichment score (NES) >1.5 and nominal p value <0.05 as the threshold [21]. Each analysis includes at least 1000 times permutation tests to discover significant critical biological pathways. PPI network analysis was also conducted to find the potential relationships among NOP2 and other genes in ccRCC, with the help of online STRING (https://string-db.org/) database by querying NOP2 gene in homo sapiens [22].

### Microsatellite instability (MSI), tumor neoantigen burden (TNB), and tumor mutational burden (TMB)

By means of the Spearman’s method, correlation analyses were performed to explore the associations among the NOP2 gene expression and TMB or MSI or TNB, a [23,24]. By the R-package ‘fmsb’, each indicator was visualized by a radar map. Above-mentioned analyses were performed using the Sangerbox tools (http://www.sangerbox.com/tool), a free online platform for data analysis by querying NOP2 gene in single-gene pancancer analysis tool [25,26].

### Tumor microenvironment, tumor immune infiltration, immune checkpoint molecules, and immune cells pathway

By utilizing NOP2 expression matrix, we calculated the ImmuneScore, StromalScore and ESTIMATEScore by applying the ESTIMATE algorithm with P < 0.001 as cutoff values [27]. We also calculated the immune cells infiltration in ccRCC by CIBERSORT with P < 0.001 as cutoff values [28]. Co-expression analysis of NOP2 expression and immune checkpoint molecules or immune cells pathway was calculated by ‘limma’ and visualized by ‘reshape2’, ‘RColorBrewer’ R-packages. Above mentioned analyses were performed using the Sangerbox tools (http://www.sangerbox.com/tool), a free online platform for data analysis by querying NOP2 gene in single-gene pancancer analysis tool [25,26].

## Results

It was the first time for us to explore the roles of NOP2 in ccRCC. We not only analyzed NOP2 mRNA expression by qRT-PCR and ICGC dataset verification, but also verified its protein expression by CPTAC analysis and the HPA database, making our results more persuasive. Moreover, multiple elements were analyzed for NOP2 in ccRCC including mutation features, nomogram, GSEA, MSI, TMB, TNB, PPI, tumor microenvironment, tumor immune infiltration, immune cell pathway, and checkpoint molecules. Taken together, NOP2 could be a potential prognostic predictor for OS in ccRCC, related to five signaling pathways and closely associated with immunity, MSI and TMB.

### Relative expression level of NOP2 in ccRCC

**Figure 1.**
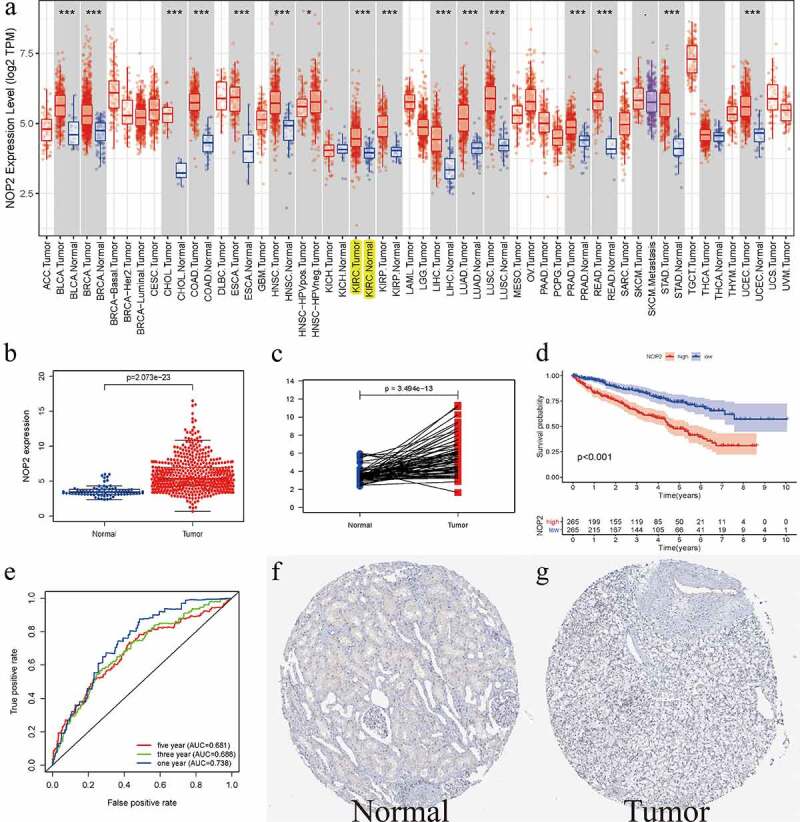
Relative expression level of NOP2 in ccRCC from TCGA and HPA database; (a) NOP2 mRNA expression levels in pan-cancers from TCGA database; (b) Boxplot of NOP2 expression between the ccRCC and normal tissues in TCGA dataset (Normal = 72 and Tumor = 539); (c) Pairwise boxplot of NOP2 expression between the ccRCC and normal tissues in TCGA dataset (Normal = 72 and Tumor = 72); (d) K–M survival analysis of NOP2; (e) ROC curves and its AUCs for 1-, 3-, and 5-year survival of NOP2; (f-g) Immunohistochemical staining from the HPA database for NOP2; *P < 0.05; ***P < 0.001

As displayed in Figure 1(a), it summarized the NOP2 mRNA expression levels in pan-cancers (33 cancer types) from TCGA database. Therein, we could easily find that NOP2 expression levels from tumor samples were much higher than its expression in normal samples including ccRCC (*P* < 0.001). Boxplot from TCGA ccRCC dataset further indicated that NOP2 had a high expression in ccRCC tumors, compared with normal kidney tissues (*P* = 3.494e-13, Normal = 72 and Tumor = 539, Figure 1(b)). Pairwise boxplot remained the same results (*P* = 2.073e-23, Normal = 72 and Tumor = 72, Figure 1(c)). According to the NOP2’s median expression, K–M survival analysis showed ccRCC patients in high-NOP2 groups had a much worse OS than those in low-NOP2 groups (*P* < 0.001, Figure 1(d)). Receiver operating characteristic (ROC) curves associated with the area under the curve (AUC) values for 1-, 3-, and 5-year survival were 0.738, 0.688, 0.681, respectively (Figure 1(e)). Moreover, immunohistochemical staining from the HPA database for NOP2 in normal kidney tissue was medium, whereas it was not detected in tumor tissue (figure 1(f-g)). We further verified the expression of NOP2 in the ICGC dataset and found that it also had a high expression in tumor tissues, compared with normal tissues (*P* = 5.062e-15; Normal = 45 and Tumor = 91; Figure S1).

### qRT-PCR results and total protein or phosphoprotein expression of NOP2 in ccRCC by CPTAC analysis

**Figure 2.**
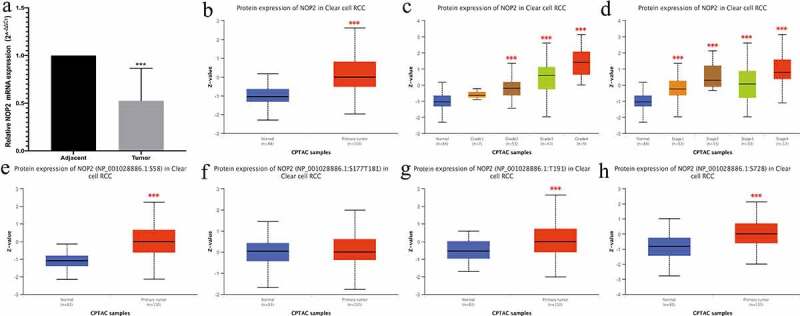
qRT-PCR results and total protein or phosphoprotein expression of NOP2 in ccRCC by CPTAC analysis; (a) qRT-PCR results of NOP2 mRNA expression in ccRCC primary tumor (N = 10) and adjacent normal tissues (N = 10); (b) Total NOP2 protein expression distribution in ccRCC primary tumor and normal tissues; (c) Total NOP2 protein expression distribution in different grades; (d) Total NOP2 protein expression distribution in different stages; (e) Expression of NOP2 phosphoprotein with site at S58 in ccRCC primary tumor and normal tissues; (f) Expression of NOP2 phosphoprotein with site at S177T181 in ccRCC primary tumor and normal tissues; (g) Expression of NOP2 phosphoprotein with site at T191 in ccRCC primary tumor and normal tissues; (h) Expression of NOP2 phosphoprotein with site at S728 in ccRCC primary tumor and normal tissues; ***P < 0.001

We utilized qRT-PCR to validate the NOP2 mRNA expression in ccRCC and found the NOP2 mRNA was down-regulated in the ccRCC tumors (N = 10) compared with normal kidney tissues (N = 10; *P* < 0.001; Figure 2(a)). We also utilized the UALCAN website to validate the NOP2 total protein and phosphoprotein expression by CPTAC analysis. As displayed in Figure 2(b), it showed the NOP2 total protein had a high expression in primary ccRCC tumors, compared with normal kidney tissues (*P* < 0.001). Figure 2(c-d) presented the NOP2 total protein expression distribution in different grades or stages, respectively. As for phosphorylation sites at the S58, S177T181, T191 and S728, NOP2 phosphoprotein expression was much higher in primary ccRCC tumors than in normal kidney tissues (all *P* < 0.001, except for S177T181, Figure 2(e-h)).

### Relationships between NOP2 expression and clinicopathologic factors

**Figure 3.**
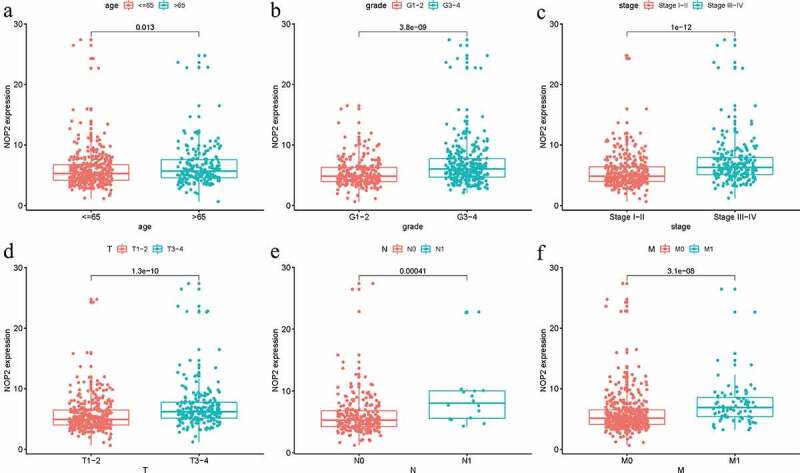
Relationships between with NOP2 expression and clinicopathologic characteristics; (a) Age; (b) Grade; (c) Stage; (d) T stage; (e) N stage; (f) M stage

Logistic regression analysis was utilized to assess the relationships among NOP2 expression and eight clinical characteristics (age, grade, stage, gender, race, T stage, M stage, N stage) in ccRCC patients from TCGA dataset. There were significant associations between high NOP2 expression and age (*P* = 0.013), grade (*P* = 3.8e-09), stage (*P* = 1e-12), T stage (*P* = 1.3e-10), M stage (*P* = 3.1e-08) and N stage (*P* = 0.00041) (Figure 3). As a result, ccRCC patients with elevated NOP2 expression were easily associated with cancer progression.

### NOP2 could be an independent prognostic factor for ccRCC

**Figure 4.**
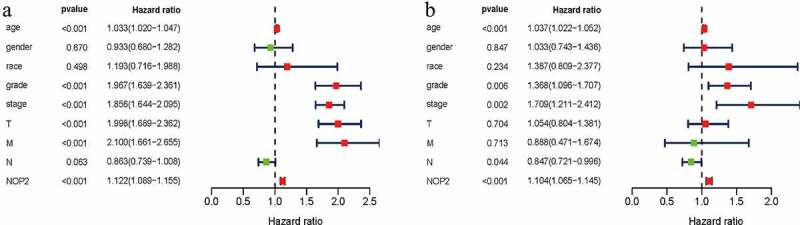
NOP2 could serve as an independent prognostic factor of OS in ccRCC; (a) Univariate Cox hazard regression analysis of clinicopathologic variables and NOP2 of ccRCC in TCGA cohort; (b) Multivariate Cox hazard regression analysis of clinicopathologic variables and NOP2 of ccRCC in TCGA cohort

Univariate Cox hazard regression analysis indicated that stage, age, grade, M stage, T stage and the NOP2 expression were all dramatically related to OS for ccRCC (all *P* < 0.05; Figure 4(a) and Table 1). Multivariate Cox hazard regression analysis indicated that age, grade, stage, N stage and the NOP2 expression were all significantly related to OS in ccRCC(all *P* < 0.05; Figure 4(b) and Table 1). Therefore, our results shed light on that the NOP2 expression, age, stage and grade all could serve as independent prognostic factors of OS in ccRCC.

**Table 1.** Univariate and multivariate analyses of NOP2 and clinicopathologic characteristics associated with OS in ccRCC patients from TCGA

Clinical characteristics	Univariate analysis				Multivariate analysis			
	HR	HR.95 L	HR.95 H	pvalue	HR	HR.95 L	HR.95 H	pvalue
**age**	1.033274	1.019678	1.047052	**1.28E-06**	1.036798	1.021576	1.052247	**1.68E-06**
**gender**	0.933298	0.679692	1.28153	0.669603	1.033036	0.743314	1.435683	0.846532
**race**	1.193075	0.71596	1.988138	0.498059	1.386993	0.809235	2.377244	0.234044
**grade**	1.966884	1.638836	2.360598	**3.70E-13**	1.368137	1.096394	1.707232	**0.005528**
**stage**	1.855626	1.643637	2.094956	**1.71E-23**	1.709158	1.211051	2.412137	**0.002293**
**T**	1.997582	1.689052	2.362469	**6.29E-16**	1.053807	0.804151	1.380971	0.70401
**M**	2.099647	1.660681	2.654644	**5.70E-10**	0.887834	0.470777	1.674355	0.713202
**N**	0.862971	0.73887	1.007916	0.062826	0.847305	0.720988	0.995754	**0.04426**
**NOP2**	1.121542	1.088897	1.155165	**2.72E-14**	1.104095	1.06502	1.144603	**7.18E-08**

### Nomogram construction based on NOP2 and clinicopathologic variables

**Figure 5.**
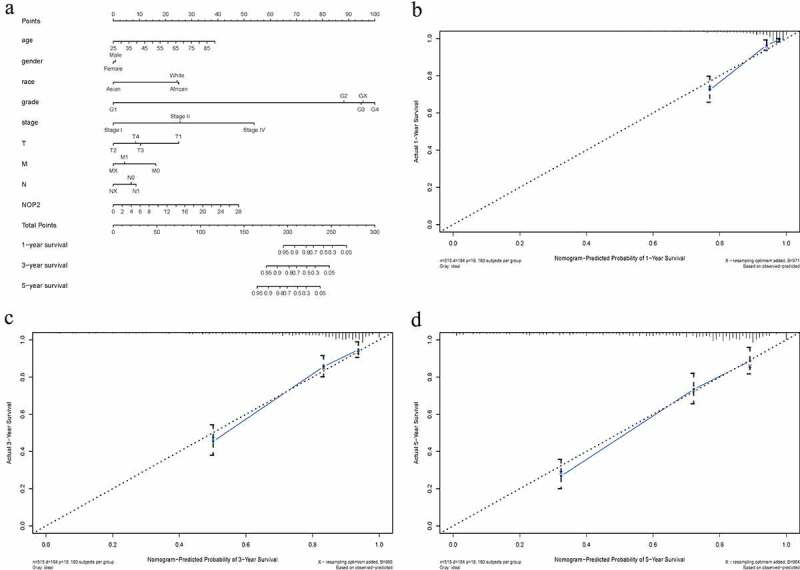
Nomogram construction and evaluation; (a) Nomogram construction based on NOP2 and clinicopathologic variables; (b) Calibration curves of 1-year; (c) Calibration curves of 3-year; (d) Calibration curves of 5-year

From this nomogram, we could obtain the total points and estimate the ccRCC patients’ survival rates at 1-, 3-, and 5-years, making the predictive method more intuitive (Figure 5(a)). Moreover, C-index and 1-, 3-, 5-year AUCs of this nomogram were 0.788, 0.857, 0.804 and 0.779, indicating a moderate prediction accuracy (Table 2). Calibration curves of 1-, 3-, and 5-years (Figure 5(b-d)) further showed a satisfactory performance for our constructed nomogram.

**Table 2.** C-index and AUCs of the constructed nomogram

	1-year	3-year	5-year	C-index
AUC	0.857	0.804	0.779	0.788

### GSEA identified NOP2-related signaling pathways

**Figure 6.**
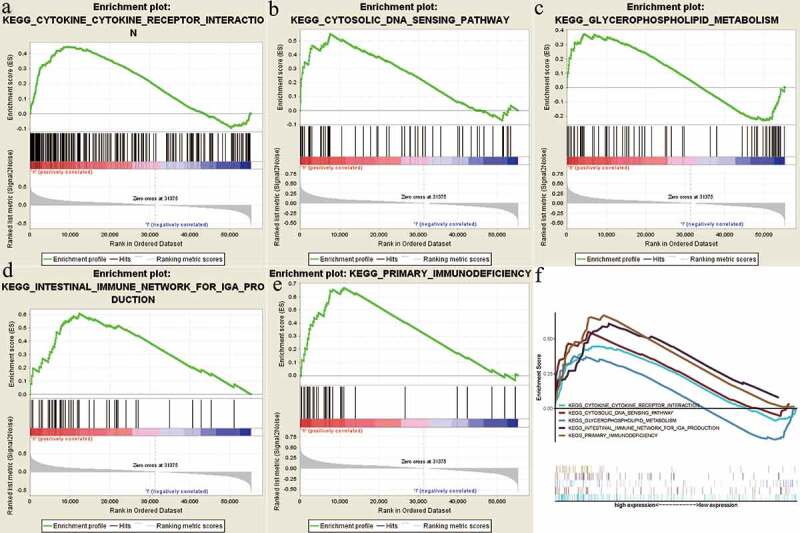
Enrichment plots from gene set enrichment analysis (GSEA); (a) Cytokine-cytokine receptor interaction pathway; (b) Cytosolic DNA sensing pathway; (c) Glycerophospholipid metabolism pathway; (d) Intestinal immune network for IgA production pathway; (e) Primary immunodeficiency pathway; (f) All of these five eligible signaling pathways significantly enriched in the high-NOP2 expression phenotype

In order to identify NOP2-related signaling pathways, GSEA was performed between high-NOP2 groups and low-NOP2 groups, with the consideration of the nominal p value <0.05 and normalized enrichment score (NES) >1.5 as the threshold. As displayed in Figure 6 and Table 3, five eligible signaling pathways exhibiting significant enrichment in the high-NOP2 expression phenotype were finally identified, containing cytokine-cytokine receptor interaction pathway, Cytosolic DNA sensing pathway, Glycerophospholipid metabolism pathway, Primary immunodeficiency pathway and Intestinal immune network for IgA production pathway. Above mentioned results might help to further understand ccRCC pathophysiological mechanisms.

**Table 3.** The results of gene set enrichment analysis

MSigDB collection	Gene set name	NES	NOM p-val	FDR q-val
c2.cp.kegg.v7.1.symbols.gmt	KEGG_CYTOKINE_CYTOKINE_RECEPTOR_INTERACTION	1.90	0.011	0.192
	KEGG_CYTOSOLIC_DNA_SENSING_PATHWAY	2.014	0.010	0.124
	KEGG_GLYCEROPHOSPHOLIPID_METABOLISM	1.637	0.008	0.326
	KEGG_INTESTINAL_IMMUNE_NETWORK_FOR_IGA_PRODUCTION	1.829	0.039	0.224
	KEGG_PRIMARY_IMMUNODEFICIENCY	1.805	0.021	0.160

### Genetic alteration analysis of NOP2 in ccRCC by cBioPortal for cancer genomics

**Figure 7.**
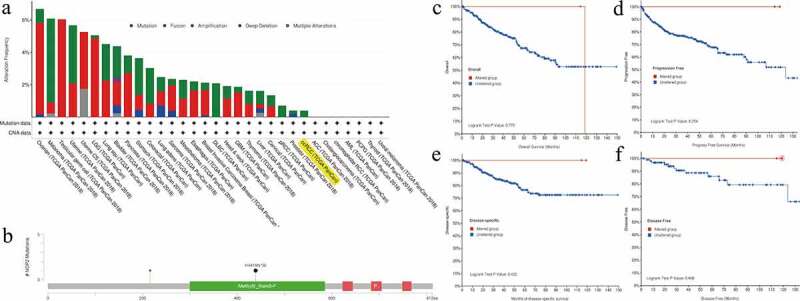
Mutation feature of NOP2 in ccRCC from TCGA cohort using the cBioPortal tool; (a) The alteration frequency with mutation type of NOP2 in different tumor samples from TCGA cohorts; (b) Mutation sites of NOP2 in ccRCC from TCGA cohort; (c) K-M survival analysis of OS with or without NOP2 alteration; (d) K-M survival analysis of progression-free survival with or without NOP2 alteration; (e) K-M survival analysis of disease-specific survival with or without NOP2 alteration; (f) K-M survival analysis of disease-free survival with or without NOP2 alteration

We utilized the cBioPortal tool to explore the mutation features of NOP2 in ccRCC from TCGA cohort and noticed that the genetic alteration frequency of NOP2 was less than 1% in ccRCC (Figure 7(a)). Mutation sites of NOP2 in ccRCC were displayed in Figure 7(b). As displayed in Figure 7(c-f), these indicated that ccRCC cases with altered NOP2 did not show better prognosis in OS (*P* = 0.779), progression-free survival (*P* = 0.254), disease-specific survival (*P* = 0.422) and disease-free survival (*P* = 0.498), compared with cases without NOP2 alteration. All in all, genetic alteration of NOP2 might not play a vital role in ccRCC.

### Relationships between NOP2 and PPI, MSI, TNB, TMB in ccRCC

**Figure 8.**
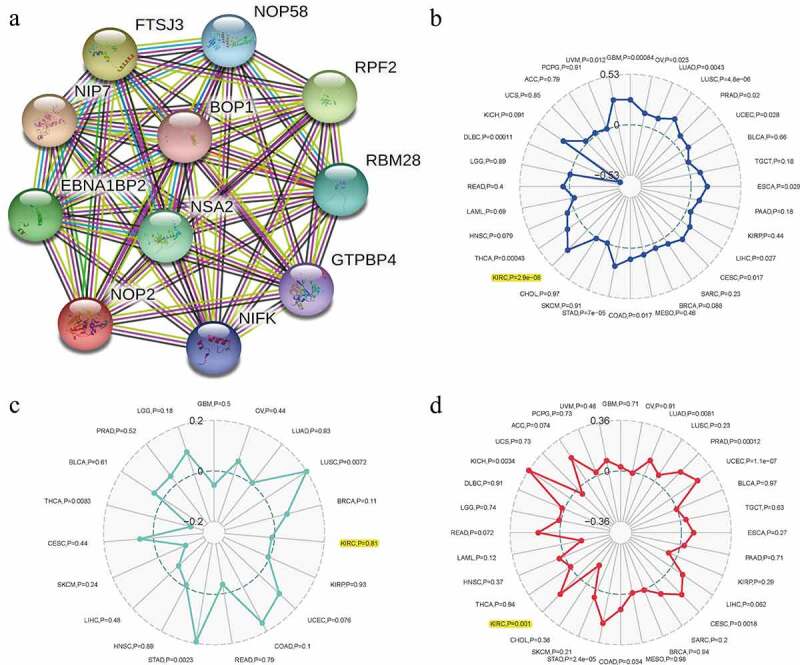
Relationships between NOP2 and PPI, MSI, TNB, TMB in ccRCC; (a) PPI network; (b) Relationships between NOP2 and MSI; (c) Relationships between NOP2 and TNB; (d) Relationships between NOP2 and TMB

To find the potential relationships among NOP2 and other genes in ccRCC, PPI network analysis was conducted with the help of online STRING (https://string-db.org/) database (Figure 8(a)). To explore the relationships between NOP2 gene expression and MSI or TMB or TNB, a correlation analysis was performed and visualized by a radar map. As displayed in Figure 8(b-d), the outcomes of us shed light on that NOP2 gene expression was significantly associated with MSI (*P* = 2.9e-08) and TMB (*P* = 0.001) in ccRCC; however, it was not related to TNB (*P* = 0.81).

### Relationships between NOP2 and tumor microenvironment, tumor immune infiltration, immune cells pathway, immune checkpoint molecules in ccRCC

**Figure 9.**
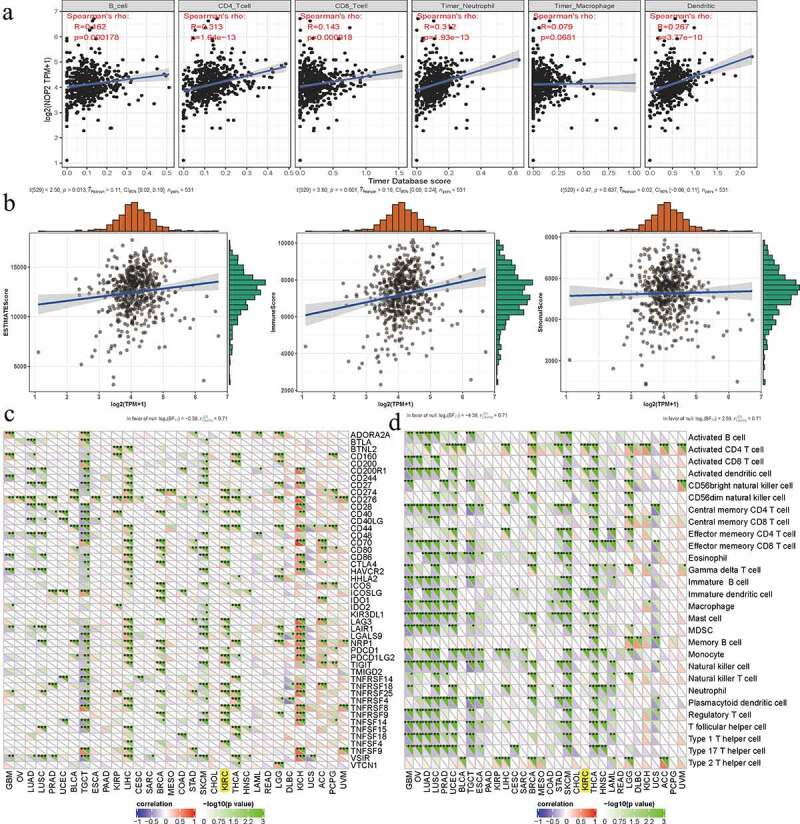
Relationships between NOP2 and tumor immune infiltration, tumor microenvironment, immune checkpoint molecules, immune cells pathway in ccRCC; (a) Relationships between NOP2 and tumor immune infiltration in ccRCC; (b) Relationships between NOP2 and immune microenvironment in ccRCC; (c) Co-expression analysis of NOP2 and immune checkpoint molecules in ccRCC; (d) Co-expression analysis of NOP2 and immune cells pathway in ccRCC

To further explore the potential relationships between NOP2 and immunity, four aspects were analyzed including tumor microenvironment, tumor immune infiltration, immune cell pathway, and immune checkpoint molecules. As for tumor immune infiltration, NOP2 was significantly associated with CD4 + T cells, B cells, neutrophils cells, CD8 + T cells and dendritic cell infiltration (all *P* < 0.001, Figure 9(a)). In terms of tumor microenvironment, NOP2 was markedly related to ImmuneScore and ESTIMATEScore (both *P* < 0.05); however, it was not linked to StromalScore (*P* = 0.637, Figure 9(b)). Co-expression analysis among immune checkpoint molecules and NOP2 presented that NOP2 was markedly related to PDCD1 (PD1), CTLA4, CD274 (PDL1), LAG3, etc., in ccRCC from TCGA dataset (all *P* < 0.05, Figure 9(c)). Co-expression analysis of NOP2 and immune cell pathways indicated that NOP2 was significantly associated with Activated CD4 T cells pathway, Activated dendritic cell pathway, Immature dendritic cell pathway, mast cell pathway, and monocyte pathway (all *P* < 0.05, Figure 9(d)).

## Discussion

As an indispensable part for cancer precision medicine, biomarkers, or signatures for predicting cancer prognosis and therapeutic benefits were urgently required [29–31]. With the assistance of high-throughput sequencing and microarray technology, various prognostic biomarkers had been identified and a lot of prognostic signatures had been established [32–34]. Under biomarker guidance, meta-analysis studies of previous clinical trials reported that response rate with targeted agents had reached to 30%, higher than chemotherapy [35]. Despite these promising outcomes, ccRCC was resistant to radiotherapy or chemotherapy and nearly one-third of patients treated with partial or radical nephrectomy would eventually develop metastatic disease [36]. Hence, it was still an urgent need to explore prognostic biomarkers for ccRCC patients.

In this article, we revealed the associations between NOP2 expression and ccRCC patients’ survival by means of TCGA data mining. Our results indicated that NOP2 had a high expression in ccRCC tumors compared with normal kidney tissues and elevated NOP2 expression was linked to poor OS, having a moderate diagnostic accuracy. Moreover, logistic regression analysis indicated that NOP2 was remarkably associated with stage, age, grade, M stage, T stage, and N stage, relating to cancer progression. Univariate/multivariate Cox hazard regression analysis results also indicated that NOP2 could be an independent prognostic factor for ccRCC. Overall, the above mentioned results revealed that NOP2 might be a prognostic predictor for ccRCC with good performance. As shown by previous articles, NOP2 was reported to be highly expressed in various cancer types, and it could also promote fibroblast growth or tumor formation, influence the cell cycle, stimulate cell proliferation, increase nucleolar activity, and associate with cancer aggressiveness in vivo and in vitro [7,8]. Sun et al. shed light on that the LINC00963/miR-542-3p/NOP2 axis could act as an inducer of prostate cancer metastasis, having a diagnostic and therapeutic potential for these patients [9].

To validate the protein expression of NOP2, CPTAC analysis showed that the NOP2 had a high expression in primary ccRCC tumor than normal kidney tissues in line with its mRNA expression levels. As protein phosphorylation had been revealed to play vital roles in multiple cancers [37–39], we also analyzed the NOP2 phosphoprotein expression and found that it was highly expressed in primary ccRCC tumor than normal tissues with phosphorylation sites at the S58, T191 and S728. In order to identify NOP2-related signaling pathways, GSEA, as a useful tool, had been applied by various researchers [40,41]. Finally, five eligible signaling pathways were identified, containing cytokine-cytokine receptor interaction pathway, cytosolic DNA sensing pathway, glycerophospholipid metabolism pathway, primary immunodeficiency pathway, and intestinal immune network for IgA production pathway. All of these identified signaling pathways helped us further understand ccRCC pathophysiological mechanisms.

Genetic alterations had been found to be linked to a lot of cancers [42,43]. However, we noticed that the genetic alteration frequency of NOP2 was less than 1% in TCGA ccRCC cohort. Moreover, altered NOP2 ccRCC samples did not show a better prognosis in progression-free survival, OS, disease-free survival and disease-specific survival, compared with samples without NOP2 alteration, indicating genetic alterations of NOP2 might not play vital roles in ccRCC. As for MSI, TMB, and TNB, they played essential roles in cancer tumorigenesis and progression [44,45]. Our results shed light on that NOP2 was significantly related to MSI and TMB, while it was not linked to TNB.

Nomogram, as a predictive tool, has been widely applied to help clinical decision-making [46–48]. In our article, we created a nomogram to intuitively predict OS probabilities in ccRCC, by means of NOP2 and eight clinical parameters (gender, age, grade, stage, T stage, race, N stage, M stage). C-index, 1-, 3-, 5-year AUCs and calibration curves indicated that this nomogram had moderate prediction accuracy and satisfactory performance. All in all, we successfully established an NOP2-based nomogram and it was anticipated to guide the prognosis of ccRCC patients.

We further explored the potential relationships between NOP2 and immunity mainly including four aspects (tumor microenvironment, tumor immune infiltration, immune cell pathway, and immune checkpoint molecules). As reported by previous articles, tumor microenvironment as well as tumor immune infiltration was associated with ccRCC prognosis and response to immunotherapy [49,50] and various genes had been found to be significantly associated with immune cells pathways and checkpoint molecules [51,52]. As for tumor immune infiltration, NOP2 was significantly associated with CD4 + T cells, B cells, neutrophil cells, CD8 + T cells and dendritic cells infiltration. In terms of tumor microenvironment, it was noticeably related to ImmuneScore and ESTIMATEScore. Co-expression analysis of NOP2 and immune checkpoint molecules or immune cell pathways presented that this gene was significantly related to CD274 (PDL1), PDCD1 (PD1), CTLA4, LAG3, Actived CD4 T cells pathway, activated dendritic cell pathway, immature dendritic cell pathway, mast cell pathway, and monocyte pathway in ccRCC from TCGA dataset. All of these indicated that NOP2 was closely related to immunity in ccRCC.

The strength of this article was that it was the first time for us to explore the roles of NOP2 in ccRCC. We not only analyzed NOP2 mRNA expression by qRT-PCR and ICGC dataset validation, but also verified its protein expression by CPTAC analysis and the HPA database, making our results more persuasive. Moreover, multiple elements were analyzed for NOP2 in ccRCC including mutation features, nomogram, GSEA, MSI, TMB, TNB, PPI, tumor microenvironment, tumor immune infiltration, immune cell pathway and checkpoint molecules. Taken together, NOP2 could be a potential prognostic predictor for ccRCC, related to five signaling pathways and closely associated with immunity. However, several limitations should not be ignored before fully understanding this article. Firstly, due to the retrospective data, clinical information from TCGA was limited and various vital data could not be obtained including underlying chronic disease, use of immunotherapy, recurrence after nephrectomy, etc. Secondly, we noticed that NOP2 was up-regulated in ccRCC tissues by bioinformatics analysis. However, qRT-PCR verification results and immunohistochemical staining from the HPA database indicated that NOP2 mRNA and protein had a low expression in ccRCC tumors. As a result, more clinical samples were required by us to verify its expression and our subsequent experiments would pay attention to verifying its potential mechanisms of NOP2 in ccRCC at both cellular and molecular levels. Thirdly, the sample sizes of normal renal tissue samples (N = 72) in TCGA were relatively small and this could lead to some biases.

## Conclusions

In summary, our outcomes shed light on that NOP2 could serve as a potential prognostic predictor of OS for ccRCC and five NOP2-related signaling pathways were identified, containing cytokine-cytokine receptor interaction pathway, cytosolic DNA sensing pathway, glycerophospholipid metabolism pathway, primary immunodeficiency pathway, and intestinal immune network for IgA production pathway. Moreover, NOP2 was dramatically linked to MSI, TMB, and immunity. Obviously, our results were expected to provide novel insights of ccRCC tumorigenesis for future work. More data supporting from clinical patients were required to further verify our findings.

## Supplementary Material

Supplemental Material

## Data Availability

The RNA-sequencing data and corresponding clinical information were downloaded from the Cancer Genome Atlas (TCGA) database (https://portal.gdc.cancer.gov/).

## Highlights

- It was the first time for us to explore the roles of NOP2 in ccRCC.
- NOP2 could be an independent prognostic factor for ccRCC patients.
- NOP2 might be a potential prognostic factor for OS in ccRCC patients.
- NOP2 was significantly associated with immunity, MSI and TMB for ccRCC.
- GSEA identified five NOP2‑related signaling pathways.
