# Supplementary figures and images for: Nucleolar protein NOP2 could serve as a potential prognostic predictor for clear cell renal cell carcinoma
Source: Bioengineered. 2021 Aug 1;12(1):4841–55. doi: 10.1080/21655979.2021.1960130 (PMC8806646; doi:10.1080/21655979.2021.1960130)

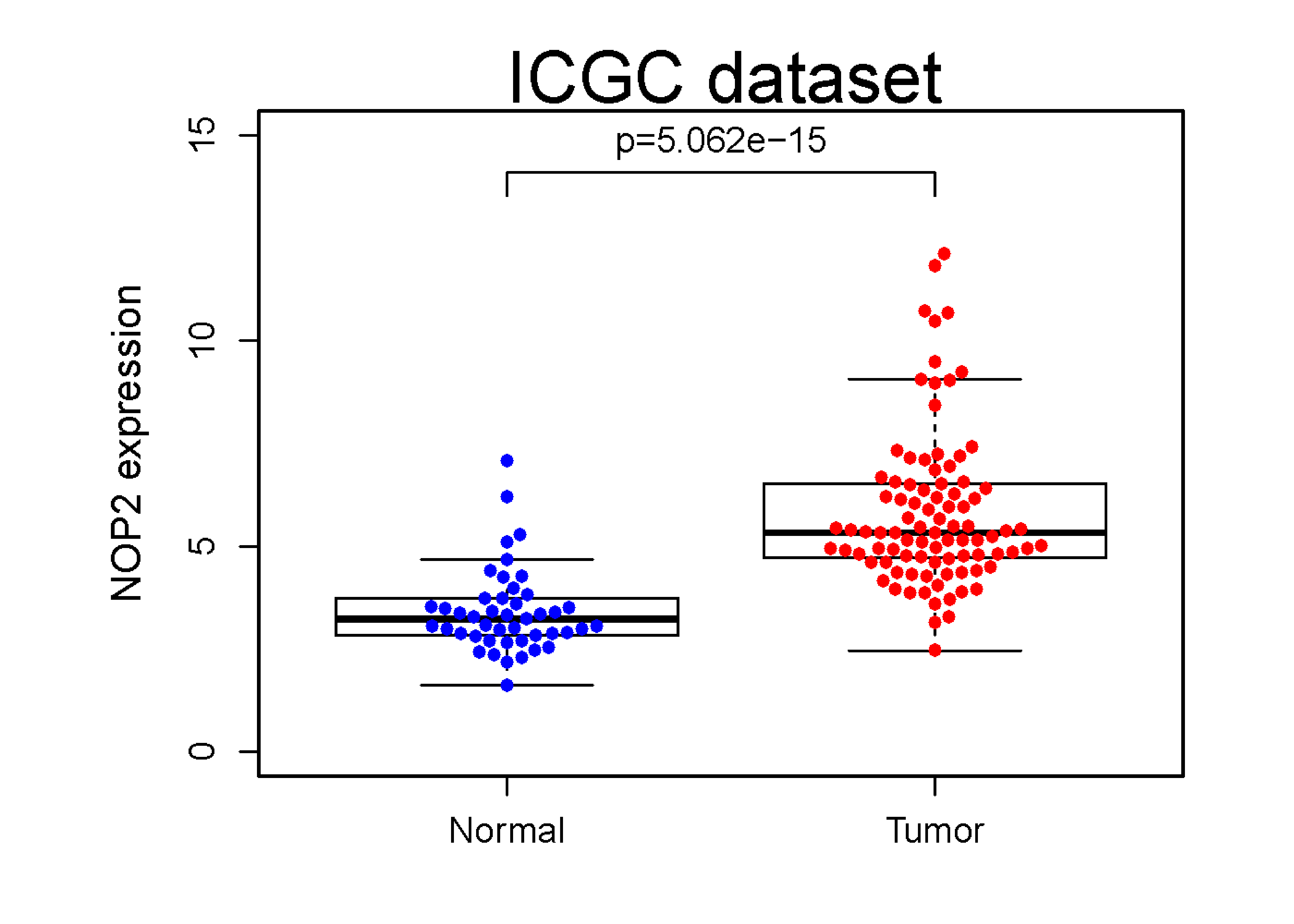

Supplement: Supplemental Material [file KBIE_A_1960130_SM3170.zip › Figure S1.tif]
